# Supplementary material for: Alanine-Scanning Mutagenesis of α-Conotoxin GI Reveals the Residues Crucial for Activity at the Muscle Acetylcholine Receptor
Source: Mar Drugs. 2018 Dec 13;16(12):507. doi: 10.3390/md16120507 (PMC6315591; doi:10.3390/md16120507)
Supplement: Supplementary file 1 [file marinedrugs-16-00507-s001.pdf]

Supplementary Information

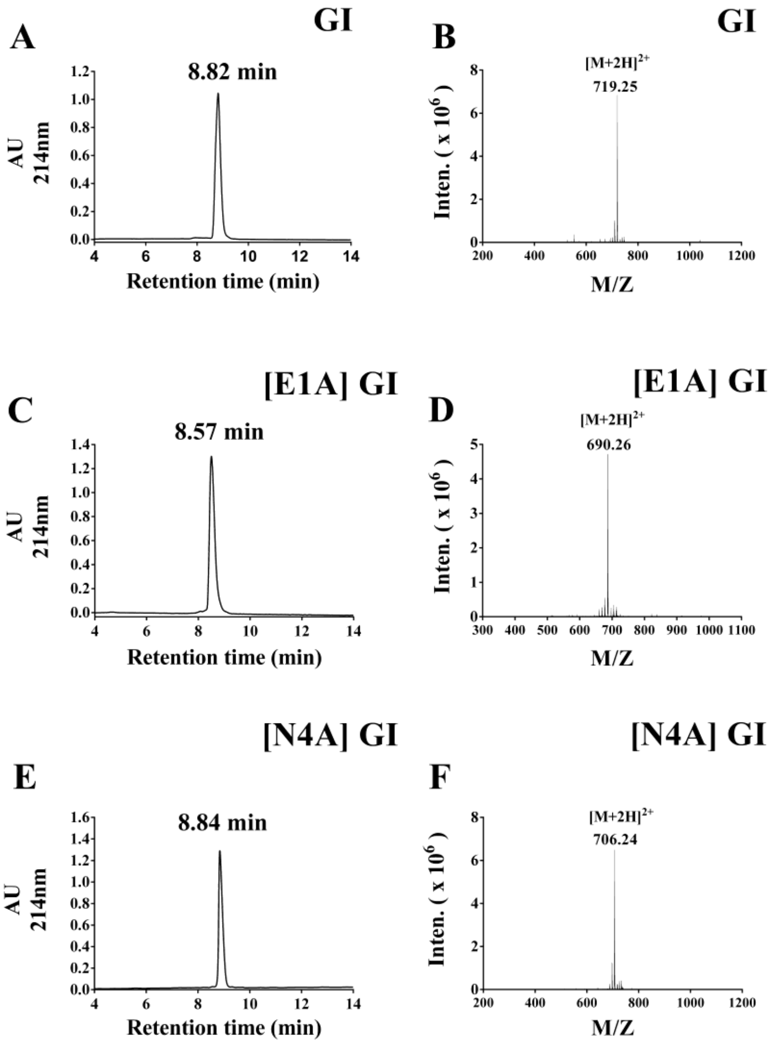

Figure S1. The HPLC chromatograms and mass spectrum of GI, [E1A] GI and [N4A] GI respectively. (A) HPLC chromatograms of GI and the retention of GI is 8.82 min; (B) electrospray ionization mass spectrometry (ESI-MS) data for GI with an observed monoisotopic mass of 1436.50 Da. (C) HPLC chromatograms of [E1A] GI and the retention of [E1A] GI is 8.54 min; (D) electrospray ionization mass spectrometry (ESI-MS) data for [E1A] GI with an observed monoisotopic mass of 1378.50 Da. (E) HPLC chromatograms of [N4A] GI and the retention of [N4A] GI is 8.84 min; (F) electrospray ionization mass spectrometry (ESI-MS) data for [N4A] GI with an observed monoisotopic mass of 1393.50 Da.

Formatted: Font: Not Bold

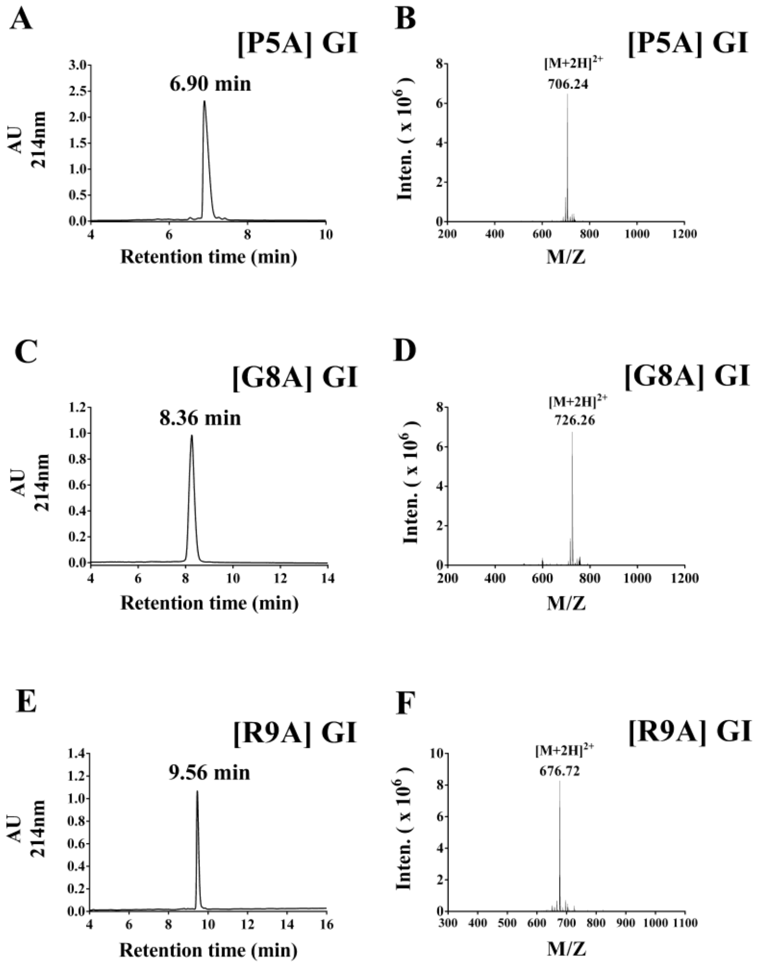

Figure 2S. ~~the~~ The HPLC chromatograms and mass spectrum of [P5A] GI, [G8A] GI and [R9A] GI respectively. (A) HPLC chromatograms of [P5A] GI and the retention of [P5A] GI is 6.90 min; (B) electrospray ionization mass spectrometry (ESI-MS) data for [P5A] GI with an observed monoisotopic mass of 1410.48 Da. (C) HPLC chromatograms of [G8A] GI and the retention of [G8A] GI is 8.36 min; (D) electrospray ionization mass spectrometry (ESI-MS) data for [G8A] GI with an observed monoisotopic mass of 1450.52 Da. (E) HPLC chromatograms of [R9A] GI and the retention of [R9A] GI is 9.56 min; (F) electrospray ionization mass spectrometry (ESI-MS) data for [R9A] GI with an observed monoisotopic mass of 1351.44 Da.

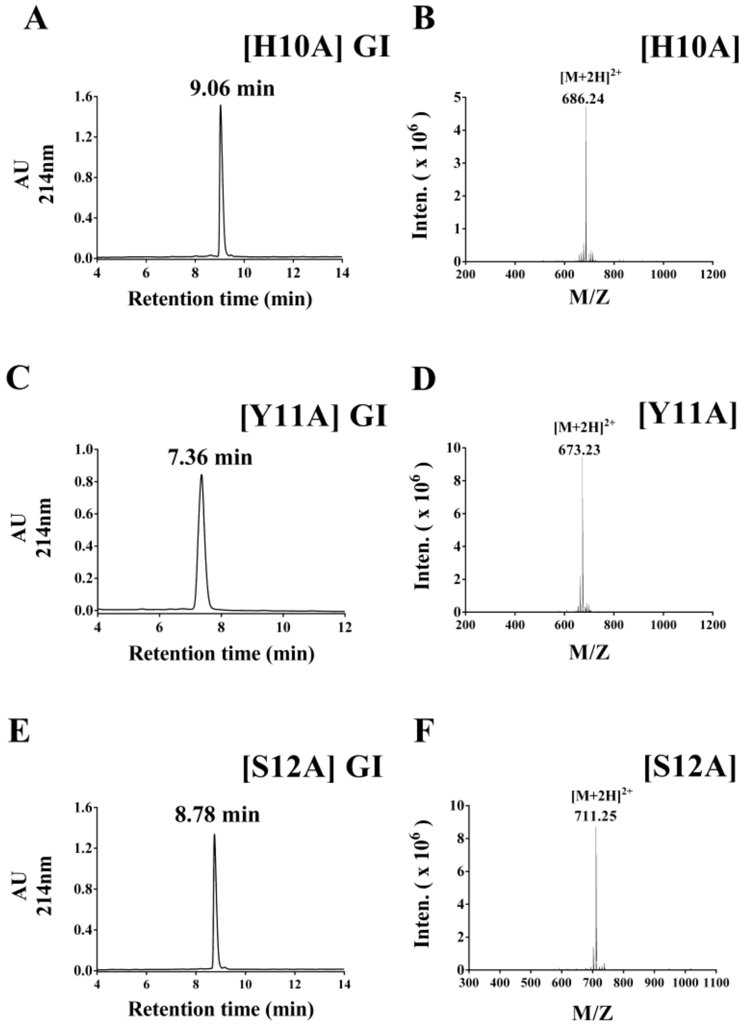

Figure 3S. The HPLC chromatograms and mass spectrum of [H10A] GI, [Y11A] GI and [S12A] GI respectively. (A) HPLC chromatograms of [H10A] GI and the retention of [H10A] GI is 9.06 min; (B) electrospray ionization mass spectrometry (ESI-MS) data for [H10A] GI with an observed monoisotopic mass of 1370.48 Da. (C) HPLC chromatograms of [Y11A] GI and the retention of [Y11A] GI is 7.36 min; (D) electrospray ionization mass spectrometry (ESI-MS) data for [Y11A] GI with an observed monoisotopic mass of 1344.47 Da. (E) HPLC chromatograms of [S12A] GI and the retention of [S12A] GI is 8.78 min; (F) electrospray ionization mass spectrometry (ESI-MS) data for [S12A] GI with an observed monoisotopic mass of 1420.50 Da.
